# Seroprevalence and risk factors associated with toxoplasmosis in nomadic, rural, and urban communities of northwestern Iran

**DOI:** 10.3389/fpubh.2025.1516693

**Published:** 2025-04-09

**Authors:** Ali Bahadori, Towhid Babazadeh, Khalil Maleki Chollou, Hanane Moqadam, Mostafa Bafandeh Zendeh, Behnaz Valipour, Leili Valizadeh, Soghra Valizadeh, Sakhavat Abolhasani, Hamed Behniafar

**Affiliations:** ^1^Department of Medical Microbiology, Sarab Faculty of Medical Sciences, Sarab, Iran; ^2^Department of Public Health, Sarab Faculty of Medical Sciences, Sarab, Iran; ^3^Department of Nursing, Sarab Faculty of Medical Sciences, Sarab, Iran; ^4^Student Research Committee, Sarab Faculty of Medical Sciences, Sarab, Iran; ^5^Department of Anatomical Sciences, Sarab Faculty of Medical Sciences, Sarab, Iran; ^6^Department of Cardiology, School of Medicine, Ardabil University of Medical Sciences, Ardabil, Iran; ^7^Department of Food Hygiene and Aquatic, Faculty of Veterinary Medicine, University of Tabriz, Tabriz, Iran; ^8^Department of Clinical Biochemistry, Sarab Faculty of Medical Sciences, Sarab, Iran; ^9^Department of Medical Parasitology, Sarab Faculty of Medical Sciences, Sarab, Iran

**Keywords:** toxoplasmosis, Iran, Seroprevalence, risk factors, ELISA

## Abstract

**Objectives:**

Toxoplasmosis is an infection that is widespread in populations comprising humans and other warm-blooded creatures and is caused by the protozoan parasite called *Toxoplasma gondii*. Hence, knowledge of seroprevalence and associated risk factors is essential for planning adequate and efficient population health interventions. The objective of the present study was to evaluate the seropositivity of *T. gondii* infection and to study the epidemiological indices in different categories of populations in East Azerbaijan Province, Iran.

**Methods:**

This cross-sectional survey involved 426 participants 10 years of age and older from urban, rural, and nomadic areas. Serum samples were collected and analyzed for IgG antibodies against *T. gondii* using the ELISA method. Demographic data, such as age, occupation, soil contact, and cat ownership, were collected through questionnaires.

**Results:**

The overall prevalence of toxoplasmosis was 62.2%, and a significant relationship between the infection and age, exposure to soil, and job involvement was observed. Farmers and ranchers had the highest occurrence rate of 69.4%; no association of the prevalence with gender, education, income, cat ownership, or eating habits was observed.

**Conclusion:**

Occupational risk factors appear to play a significant role in the transmission of toxoplasmosis, although there is doubt cast on classic risk factors such as cat handling and ingesting contaminated foodstuffs. Such exposure can lead to risky transmission of *T. gondii*; therefore, continued surveillance and specific approaches in public health are needed to address such risks in the area.

## 1 Introduction

Toxoplasmosis is a Protozoan disease caused by the intracellular protozoan parasite *Toxoplasma gondii* that can infect humans and various warm-blooded animals. Toxoplasmosis is a highly prevalent infection, and studies have estimated that as many as one-third of the adult worldwide population may be infected with the parasite (1). Despite the high global prevalence, the exact prevalence within any human community is significantly influenced by factors such as geography, climate, dietary habits, and levels of sanitation (2). Felidae species, both pets and wild, are acknowledged as the unique definitive hosts of this parasite.

Transmission primarily occurs via foodborne pathways, including ingesting oocyst-contaminated food or water and consuming raw or undercooked meat harboring tissue cysts. Congenital transmission also poses significant risks, particularly to fetuses (3). The parasite can accomplish its life cycle by transferring from warm-blooded intermediate hosts to the cat as the final host (3, 4). In immunocompetent adults, *T. gondii* infection is typically asymptomatic or mild, with 10–30% of cases showing symptoms like lymphadenopathy. However, in immunocompromised individuals, it can lead to severe outcomes, including central nervous system (CNS) disorders (5). Moreover, vertical transmission can result in severe consequences like seizures and cognitive impairments in newborns (5). Additionally, recent studies have indicated that latent toxoplasmosis may be considered a significant contributor to various psychotic conditions, such as Parkinson’s disease, Alzheimer’s disease, bipolar disorder, and schizophrenia (6, 7).

Globally, studies report seroprevalence rates from 20% in younger adults to 77% in older populations, reflecting cumulative exposure (8). Occupational exposure, particularly among farmers, and environmental factors such as soil contact are consistently identified as risk factors (8–10). Conversely, traditional risk factors such as cat ownership and raw meat consumption exhibit inconsistent associations across different populations (11, 12). Studies conducted since 2020 have enhanced our understanding of *T. gondii* epidemiology in Iran. Hamidi et al. synthesized data from 2000 to 2023, highlighting higher rates in rural settings (13). Hassanen et al. (14) investigated cat-human transmission dynamics, while Maleki et al. (10) identified soil as a key reservoir. Also, a systematic review and meta-analysis study estimated a 45.1% prevalence of toxoplasmosis among immunocompromised individuals in Iran (15).

In Iran, toxoplasmosis is a public health concern, and previous research indicates significant variability in seroprevalence, with the average prevalence ranging from 12.5 to 61% in different provinces (13). East Azerbaijan Province in northwestern Iran, with its diverse nomadic, rural, and urban populations, has been the focus of numerous epidemiological studies from 2000 to 2023, reporting provincial rates of around 46% (16). However, the most recent study in East Azerbaijan Province was conducted in 2018 and was limited to Tabriz city (16). Also, the mentioned study did not aim to find risk factors in the general population and focused on the relationship between toxoplasmosis and diabetes. Consequently, a study extending across the whole had to be conducted in the present time to obtain accurate and detailed data; therefore, this study aims to comprehensively understand the seroprevalence and risk factors of toxoplasmosis across the entire East Azerbaijan Province, including urban, rural, and nomadic populations. By doing so, it seeks to identify variations in infection rates and associated risk factors among these different demographic groups, which have not been previously explored in such detail. This approach will contribute valuable insights into the epidemiology of toxoplasmosis in this region and inform targeted public health interventions, building on recent findings such as Arefkhah et al. (17) on nomadic communities in southwestern Iran. It is worth noting that this study uniquely investigates *T. gondii* across diverse settings, including underrepresented nomadic communities.

## 2 Methods

### 2.1 Study area

East Azerbaijan Province is located in Northwestern Iran, specifically at 37.7512° latitude North and 45.8675° longitude East; it occupies an area of about 45,491 square Kilometer. The administrative capital, Tabriz, is located approximately 130 KM from the Caspian Sea (32). Geographically, East Azerbaijan borders West Azerbaijan to the west, Ardabil Province to the north, the Republic of Azerbaijan to the northwest, the Nakhchivan Autonomous Republic to the northwest, and Armenia to the north. According to the 2016 census, the human population in the province is approximately 3,909,652. The province is divided into twenty-one counties: Tabriz, Ahar, Ajabshir, Azarshahr, Bostanabad, Bonab, Charuymaq, Hashtrood, Heris, Horand, Jolfa Kaleybar, Khod-Afarin, Malekan, Maragheh, Marand, Mianeh, Oska, Sarab, Shabestar and Varzaq.

The climate in East Azerbaijan is Cold and dry; however, because the region has differentiated territory, there are various climates. The climate of the area is formed by cold northern and Siberian currents mixed with moisture currents from the Black Sea, Mediterranean Sea, and the Atlantic Ocean. Local winds developing around the high mountains and the Urmia and Caspian lakes also influence the climate of the plains and lowlands. East Azerbaijan, which tends to be semi-arid, enjoys low average annual rainfall between 250 and 300 mm and a high degree of mountainous nature.

### 2.2 Sampling

**Figure 1:**
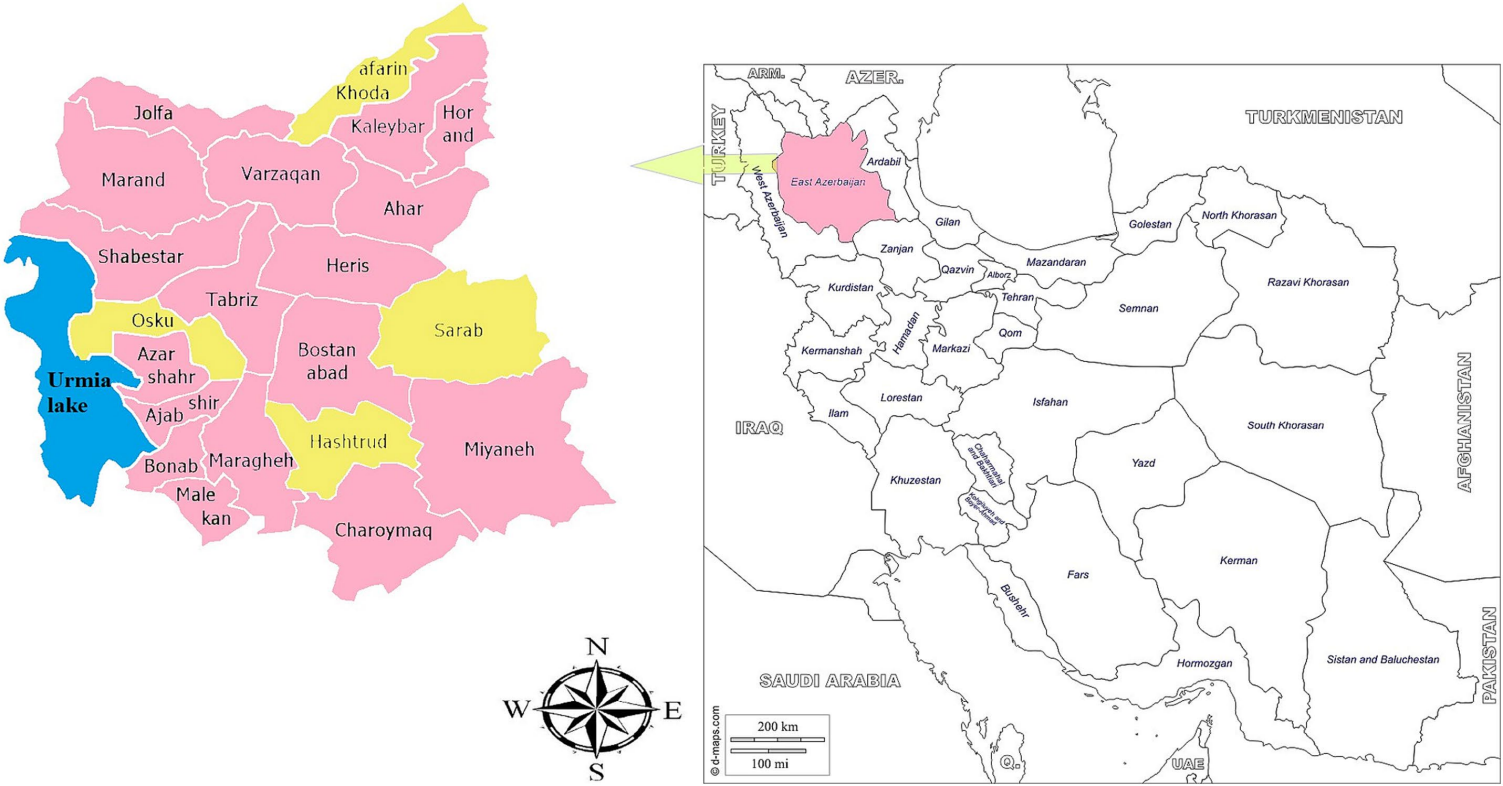
The location of East Azerbaijan Province (marked in pink) on the map of Iran and the location of the sampling counties (marked in yellow) on the map of East Azerbaijan Province.

The current cross-sectional survey is approved as IR.SARAB.REC.13402.012 was conducted to assess the seroprevalence of toxoplasmosis among participants aged 10 years and older living in urban, rural, and nomadic areas of East Azarbaijan. The sample size was calculated using the formula for estimating a population proportion (n= $\frac{z^{2}\times p\;\left(1-p\right)}{d^{2}}$ ), assuming a seroprevalence of 29.23% from a previous study, a precision of 5%, and a 96% confidence level, yielding approximately 321 participants (18). To ensure the robustness of the study, a 20% non-response rate was accounted for, increasing the target to 385. Finally, 426 participants were enrolled between June 2023 and March 2024, significantly enhancing the statistical power and accuracy of the results. Four counties, namely Khoda-Afarin, Sarab, Hashtroud, and Osku, were randomly selected in a wide geographic area for the survey, as depicted in Figure 1. While the sample size had been estimated to be 321 in the statistical calculation, 385 participants were selected to increase the reliability of the results. Prior to collecting blood samples, a questionnaire containing geographical location, age, sex, residency status (nomadic, rural, or urban), clinical symptoms, level of education, level of income, cat ownership, occupation, raw vegetable consumption, raw or undercooked meat consumption, contact with soil, and a consent form was filled out by the participants. Then, about 5 mL of blood was collected from each participant. Blood samples were centrifuged for 10 min at 1900 g; the separated sera were frozen at −20°C until serological analysis.

### 2.3 Serological test

The enzyme-linked immunosorbent assay was employed in the present study to detect serum samples against *T. gondii* for human IgG antibodies. Assays were performed in 96-well microplates. In the anti- *T. gondii* IgG assay, the PISHTAZTEB® Toxoplasma IgG ELISA KIT from Iran used in the research. This kit is based on the indirect ELISA method, and the manufacturer claims 100% sensitivity and specificity. Tests were carried out according to the instructions of the manufacturing company. Two different operators independently verified each test result for accuracy. The OD readings were recorded at a wavelength of 450 nm using an ELISA reader (Hiperion MPR4++, Germany).

### 2.4 Statistical analysis

The results were analyzed using the SPSS Statistics software, specifically version 27, which IBM developed in New York, United States. The average ± deviation was used to explain continuous variables like age, and percentages and frequencies were used for ordinal and nominal variables. It is worth mentioning that the variables include the continuous variable age, dichotomous variables including gender, cat ownership, raw vegetable consumption, raw or undercooked meat consumption, and frequent contact with soil, nominal variables including residence, occupation, and county, and ordinal variables including education and income level.

The Pearson’s chi-square (*χ*^2^) test was used to compare categorical and binary variables. Multiple logistic regression analysis was used to estimate Adjusted Odds Ratios (AORs) with 95% confidence intervals (CIs) after checking assumptions for logistic regression, such as multicollinearity and linearity between the independent variables, independence of errors, and the lack of outliers. The Hosmer and Lemeshow statistic were carried out to assess the model’s goodness of fit (19–21). The analyses were conducted using IBM SPSS Statistics Software, v. 22 (IBM, NY, United States). A *p-*value of <0.05 was considered statistically significant.

## 3 Results

### 3.1 Distribution of participants

Although 385 samples, including a 20% error, were needed, 426 serum samples were collected and tested. Most participants (*n* = 225; 52.8%) were female. Their ages ranged from 13 to 86 years old, with a mean age of 45.51 (± SD 15.97). Most participants (51.6%) fell into the middle-income bracket and were under 50 (41.8%). Most of the women (71.5%) were homemakers. Regarding education level, 129 (30.3%) participants lacked literacy. Table 1 displays socio-demographic characteristics.

**Table 1:** Assessment of risk factors for seroprevalence of *Toxoplasma* infection among nomadic, rural, and urban populations in Northwestern Iran, based on sociodemographic characteristics, using Pearson’s chi-square test.

Variables	No. of Participants (%)	No. of seropositives (%)	*p*-value
Age			<0.001
10–30	86 (20.2)	31 (36)	
31–50	162 (38)	101 (62.3)	
50<	178 (41.8)	133 (71.7)	
Gender			0.071
Female	225 (52.8)	149 (66.2)	
Male	201 (47.2)	116 (57.7)	
Residence			0.251
Urban	153 (35.9)	89 (58.2)	
Rural	256 (60.1)	163 (63.7)	
Nomadic	17 (4)	13 (76.5)	
Education			<0.001
Illiterate	129 (30.3)	102 (79.1)	
Primary and Guidance	176 (41.3)	96 (54.5)	
High school and higher	121 (28.4)	67 (55.4)	
Income level			0.057
Low	194 (45.5)	128 (66)	
Medium	220 (51.6)	133 (60.5)	
High	12 (2.8)	4 (33.3)	
Occupation			<0.001
Farmer and Rancher	98 (23)	68 (69.4)	
Student	27 (6.3)	5 (18.5)	
Clerk	48 (11.3)	22 (45.8)	
Housewife	161 (35.4)	109 (62.2)	
Unemployed	102 (23.9)	61 (59.8)	
Cat ownership			0.056
Yes	154 (36.2)	105 (68.2)	
No	272 (63.8)	160 (58.8)	
Raw vegetable consumption			0.474
Yes	389 (91.3)	244 (62.7)	
No	37 (8.7)	21 (56.8)	
Raw or undercooked meat consumption			0.574
Yes	47 (11)	31 (66)	
No	379 (89)	234 (61.7)	
Frequent contact with soil			<0.001
Yes	159 (37.3)	121 (76.1)	
No	267 (62.7)	144 (53.9)	
County			<0.001
Osku	109 (25.6)	85 (78)	
Hashtroud	104 (24.4)	73 (70.2)	
Sarab	145 (34)	53 (36.5)	
Khoda-Afarin	68 (16)	54 (79.4)	

### 3.2 Univariate analysis for risk factors of seroprevalence of anti-*T. Gondii* antibodies

It was found that 265 out of 426 individuals were seropositive. Therefore, the seroprevalence rate was calculated about 62.2%. Findings from the univariate analysis indicated that *T. gondii* seropositivity was linked to age (*p* < 0.001), education level (*p* < 0.001), job (*p* < 0.001), exposure to soil (*p* < 0.001), and location (*p* < 0.001). Complete data is provided in Table 1.

### 3.3 Logistic regression analysis for risk factors of seropositivity to *T. Gondii*

The logistic regression analysis revealed a significant association of toxoplasmosis with certain risk factors, including the age group, where increasing age increases the rate of infection; contacts with soil; and occupational categories, with the highest prevalence in farmers and ranchers, housekeeping, and unemployed individuals as opposed to other professions. On the other hand, there was no significant relationship between toxoplasmosis and other factors, including gender, type of residence, education level, income level, cat ownership, consumption of raw vegetables, consumption of raw or undercooked meat, and county of residence (Table 2).

**Table 2:** Seroprevalence of IgG antibodies to *T. gondii* according to sociodemographic characteristics estimated by multivariable logistic regression models in East Azerbaijan Province.

Variables	No. of Participants (%)	No. of seropositives (%)	*p*-value	Odds Ratio (OR)	Confidence Interval (95%)	
					Lower	Upper
Age			<0.001	2.656	1.836	3.843
10–30	86 (20.2)	31 (36)				
31–50	162 (38)	101 (62.3)				
50<	178 (41.8)	133 (71.7)				
Gender			0.066	1.521	0.973	2.376
Female	225 (52.8)	149 (66.2)				
Male	201 (47.2)	116 (57.7)				
Residence			0.501	1.167	0.745	1.829
Urban	153 (35.9)	89 (58.2)				
Rural	256 (60.1)	163 (63.7)				
Nomadic	17 (4)	13 (76.5)				
Education			0.968	1.008	0.692	1.468
Illiterate	129 (30.3)	102 (79.1)				
Primary and Guidance	176 (41.3)	96 (54.5)				
High school and higher	121 (28.4)	67 (55.4)				
Income level			0.353	0.817	0.534	1.251
Low	194 (45.5)	128 (66)				
Medium	220 (51.6)	133 (60.5)				
High	12 (2.8)	4 (33.3)				
Occupation			0.012	1.222	1.045	1.43
Farmer and Rancher	98 (23)	68 (69.4)				
Student	27 (6.3)	5 (18.5)				
Clerk	48 (11.3)	22 (45.8)				
Housewife	161 (35.4)	109 (62.2)				
Unemployed	102 (23.9)	61 (59.8)				
Cat ownership			0.204	0.702	0.406	1.212
Yes	154 (36.2)	105 (68.2)				
No	272 (63.8)	160 (58.8)				
Raw vegetable consumption			0.512	0.772	0.357	1.671
Yes	389 (91.3)	244 (62.7)				
No	37 (8.7)	21 (56.8)				
Raw or undercooked meat consumption			0.126	0.548	0.254	1.184
Yes	47 (11)	31 (66)				
No	379 (89)	234 (61.7)				
Frequent contact with soil			<0.001	0.29	0.155	0.542
Yes	159 (37.3)	121 (76.1)				
No	267 (62.7)	144 (53.9)				
County			0.528	1.09	0.84	1.426
Osku	109 (25.6)	85 (78)				
Hashtroud	104 (24.4)	73 (70.2)				
Sarab	145 (34)	53 (36.5)				
Khoda-Afarin	68 (16)	54 (79.4)				

## 4 Discussion

In this study, the serum IgG level of the participants was measured using ELISA. Since humoral immunity plays a determining role in immune reactions and protection against infections, the IgG antibody levels may be considered one of the best parameters reflecting the immunological resistance of a given subject to infections. Therefore, enzymatic immunoassay techniques such as ELISA can help assess the immune system level.

The present study is the first to investigate seroprevalence and associated risk factors of toxoplasmosis in East Azerbaijan Province since 2018 (16). Compared to the last published survey, which was only conducted in Tabriz, the present study included four cities from different geographical regions. In contrast to the previous studies, the present study includes all three demographic categories: urban, rural, and nomadic populations. Its originality lies in its inclusion of nomadic populations and broad geographic scope, and it has been updating data since 2018. Although the seroprevalence found in the present cross-sectional study is higher than the average seroprevalence in Iran (32.9%) and East Azerbaijan province (46%), it is within the range of some studies (13). For example, Khalili et al. (16) found that 59% of the healthy adult population tested positive for toxoplasmosis in 2016. In addition, Mahami et al. (22) reported a prevalence of 62% among healthy adults in Tabriz in 2016. Another study reported a prevalence of 33.5% of individuals referred to diagnostic laboratories in Markazi Province (23). These findings suggest significant variability in seroprevalence rates across different regions and populations, highlighting the need for targeted public health interventions and further research to understand the factors contributing to these differences.

In the current study, univariate analysis linked multiple variables to *T. gondii* infection (Table 1). However, in the multivariate logistic regression analysis, among the risk factors investigated, a significant association was found between toxoplasmosis and three risk factors: age, contact with soil, and occupation. Age-related increases in seropositivity align with global trends, reflecting cumulative exposure (8). Soil contact and farming occupations emerged as key risk factors, supported by recent studies (10), while traditional factors like cat ownership were non-significant, challenging earlier assumptions (11).

Seroepidemiologic studies revealed that prevalence increases with age due to increased exposure to the parasite throughout the lifespan. For example, seroprevalence increased from 20% in 18- to 29-year-olds to up to 77% in 70- to 79-year-olds, showing that infections increase with age (8). Another study found that the infection rate is highest in people over the age of 40, suggesting that this may be due to the sum of interactions with the virus (24). This age-related trend underscores the importance of targeted public health interventions aimed at sensitive groups, such as immunocompromised individuals in older ages, who may be more vulnerable to both exposure and severe outcomes associated with toxoplasmosis.

This statistical analysis showed that contact with soil increases the risk of toxoplasmosis by a large percentage, which is consistent with previous studies on environmental transmission of the *T. gondii* parasite (9, 10). According to these studies, soil can serve as a source of the parasite’s oocysts, which are excreted in the feces of sick cats. When people use contaminated soil for gardening or play outdoors, they are likely to ingest these oocysts and become infected. Implementing effective educational campaigns about safe gardening practices and hygiene can significantly reduce the risk of infection, thereby protecting vulnerable populations from potential health threats.

This study reveals a strong correlation between occupation and toxoplasmosis rates, and the data collected provides valuable information about the spread of this infection. The prevalence of toxoplasmosis among farmers and ranchers, who were expected to have the highest infection rate due to direct contact with soil, livestock, and other sources of *T. gondii* oocysts, was 69.4%. These people spend most of their time in places where they may be exposed to *T. gondii* oocysts. Previous studies have consistently found that people in agriculture often have higher seroprevalence levels for the same reasons as handling soil and animal products (25). Understanding these occupational hazards is crucial for developing targeted prevention strategies, particularly in rural areas where farming practices may inadvertently increase exposure to this parasite.

Another group with a high prevalence rate (62%) is homemakers, possibly due to gardening or handling raw meat without proper hand washing. Oocysts found in contaminated soil or on surfaces can contact with this group. Infection could be prevented if safe food handling and hygiene programs were extended to this group (26, 27).

The unemployed group, which makes up 59.8% of respondents, is interesting; they may not be occupationally contaminated, but if they have come into contact with contaminated surfaces, water, soil, or other objects due to their previous working conditions, they are more likely to handle contaminated objects or surfaces.

Clerks (45.8%) and students (18.5%) have lower prevalence rates and should presumably be exposed to conditions suitable for *T. gondii* transmission less often. In all likelihood, students may have less contact than those directly involved in some of the tasks described above. At the same time, clerks are indoor people, and their contact with soil or animals is minimal at the most (11, 26).

The findings of this study suggest that toxoplasmosis, as assessed by risk factors, is not associated with gender, household type, education level, income level, owing cats, consumption of raw vegetables, consumption of raw or rare meat, and county of residence. This result means that these commonly hypothesized risk factors may not even significantly impact the transmission patterns of *T. gondii* in the target population. For example, previous reviews have indicated that cat ownership and consumption of red and processed meat are associated with increased risk, and our findings suggest that these findings do not generalize to all populations. Several studies indicated that seroprevalence could be related to the remaining population’s direct contact with cats or raw meat consumption (11, 14, 28, 29). Several other studies did not support or hesitate the preceding by presenting varying seroprevalence results and the factors above (12, 26, 30). For instance, a study by Karimi et al. (31) supports our finding that there is no significant association with cat ownership. The absence of a significant correlation between owning a cat and toxoplasmosis suggests that cats can roam freely in urban or rural areas without being restricted to their owner’s home or suggests environmental oocyst transmission via soil may overshadow traditional risk factors in this region. Also, the lack of association between dietary (consumption of meat) and toxoplasmosis may reflect local cooking norms, meriting further investigation because a small percentage (11%) of study participants had a history of consuming raw or undercooked meat.

Moreover, the study reveals a notable absence of correlation between educational attainment and income levels, suggesting that other variables may significantly influence susceptibility to toxoplasmosis. This finding highlights the potential impact of environmental and behavioral factors that warrant further investigation. It is essential to delve deeper into these aspects to understand how they might contribute to infection rates beyond the control variables assessed in the current research. Such exploration could uncover critical insights into the multifaceted nature of toxoplasmosis susceptibility and its underlying causes, prompting a more comprehensive approach to prevention and intervention strategies.

## 5 Conclusion

In conclusion, the present study provides valuable data regarding toxoplasmosis in East Azerbaijan province to stress the necessity of continuing monitoring and control strategies to mitigate *T. gondii* exposure hazards among sensitive groups such as pregnant women.

### 5.1 Limitations

Despite obtaining valuable data concerning the seroprevalence of toxoplasmosis in East Azerbaijan province within the scope of the present study, some limitations should be considered. The cross-sectional design of this study restricts causal conclusions about the identified risk factors. Additionally, self-rating data on activities like soil contact and feeding habits may be affected by self-bias.

## Data Availability

The raw data supporting the conclusions of this article will be made available by the authors, without undue reservation.
